# An integrated explainable AI framework with counterfactual optimization for decision support

**DOI:** 10.1371/journal.pone.0358643

**Published:** 2026-09-23

**Authors:** Nusrat Jahan, Jannatul Maua, Uland Rozario, Hashibul Ahsan Shoaib

**Affiliations:** 1 Department of Business Administration and Management, Washington University of Science and Technology, Alexandria, Virginia, United States of America; 2 Department of CSE, Bangladesh University of Business and Technology, Dhaka, Bangladesh; 3 Information Technology, St. Francis College, Brooklyn, New York, United States; Institute of Management Sciences Peshawar, PAKISTAN

## Abstract

Artificial intelligence is increasingly used to support managerial decision-making in domains such as human resource management and customer relationship management; however, the lack of transparency in complex predictive models limits their practical adoption. This paper proposes a counterfactual-guided explainable artificial intelligence framework that integrates deep learning, feature attribution, and counterfactual reasoning to deliver accurate, interpretable, and actionable managerial insights. The proposed model is evaluated on an employee attrition dataset and a customer churn dataset, as well as in a combined multi-domain setting. Experimental results show that the framework achieves classification accuracies of up to 91.4% for employee attrition prediction and 87.3% for customer churn prediction, with corresponding ROC–AUC scores reaching 0.869 and 0.891, respectively. The model also demonstrates improved probability calibration, reducing the Brier score to 0.128 on the attrition dataset and 0.109 on the churn dataset. Furthermore, explainability analysis yields higher SHAP fidelity scores exceeding 0.90, while the counterfactual module generates valid and plausible recommendations with lower action costs compared to baseline approaches. These results indicate that the proposed framework effectively bridges predictive performance, interpretability, and actionability, making it suitable for real-world managerial decision-support systems. The framework is formulated as a constraint-aware optimization problem that integrates predictive modeling, explanation fidelity, and cost-sensitive counterfactual generation into a unified objective, enabling actionable and feasible decision support.

## 1 Introduction

In recent years, organizations across sectors have increasingly relied on data-driven approaches to support complex managerial decision-making processes [1]. Decisions related to employee retention, customer loyalty, risk management, and resource allocation now involve analyzing large volumes of heterogeneous data generated from organizational operations, digital platforms, and customer interactions [2]. Artificial intelligence (AI) and machine learning techniques have shown strong potential in this context by enabling accurate predictions and uncovering patterns that can guide strategic and operational decisions [3]. Empirical evidence also suggests that AI-based advisory and analytics tools can change how professionals make choices and learn better decision rules, which makes careful human-in-the-loop design important in managerial settings [4,5]. As a result, AI-based decision-support systems are becoming an integral part of modern management practices.

Despite these advances, the adoption of AI in managerial decision-making remains constrained by a critical challenge: limited transparency and interpretability in complex predictive models [6]. Many high-performing AI models, particularly deep neural networks, behave as black boxes that output predictions without exposing the underlying decision logic, which makes it difficult to justify outcomes in real operational settings [7]. In managerial contexts, this opacity creates practical risks for trust, accountability, and governance, and it complicates compliance with responsible-AI expectations in organizations [8]. Managers often need both accurate risk estimates and clear, human-understandable reasons that connect predictions to controllable business levers (e.g., retention programs, service changes, or policy interventions) [9]. This gap between predictive performance and actionable explainability continues to limit the real-world impact and adoption of AI-driven managerial analytics [10].

Motivated by these challenges, this paper addresses the problem of how to design an AI framework that simultaneously delivers strong predictive performance, transparent explanations, and actionable recommendations for managers. Existing explainable AI approaches often focus on post hoc interpretation of model outputs, while others emphasize predictive accuracy at the expense of interpretability. Moreover, many studies remain confined to a single application domain and do not consider whether an explainable framework can generalize across different managerial decision problems, such as employee attrition and customer churn. There is therefore a clear need for an integrated approach that connects prediction, explanation, and decision support in a unified and generalizable manner.

The significance of this research lies in integrating predictive modeling, explainability, and counterfactual reasoning into a unified decision-support framework for managerial applications. This integration supports transparent risk assessment, accountable decision processes, and actionable intervention planning in domains such as employee retention and customer relationship management.

From a methodological perspective, the proposed CF-XAI-DS (Counterfactual Explainable Artificial Intelligence for Decision Support) framework is formulated as a constraint-aware optimization problem that integrates predictive modeling, explanation fidelity, and cost-sensitive counterfactual generation within a unified decision-support setting. Unlike conventional XAI pipelines that treat prediction, explanation, and intervention analysis as separate stages, the proposed approach establishes an explicit linkage between these components, enabling a consistent transition from risk estimation to actionable recommendations. In particular, the framework incorporates feasibility constraints and feature-level intervention costs into the counterfactual generation process, ensuring that recommended actions remain realistic and implementable. This formulation provides a principled basis for combining interpretability and actionability in managerial decision-making contexts.

The primary objective of this research is to develop a counterfactual-guided explainable AI framework that supports managerial decision-making by answering three key questions: who is at risk, why the risk exists, and what actions can be taken to change the outcome. Specifically, the goals of this study are to:

- build a high-performing predictive model for employee attrition and customer churn,
- provide faithful and consistent explanations of model predictions using feature attribution methods,
- generate actionable counterfactual recommendations that align with realistic managerial interventions,
- evaluate the framework across multiple domains to assess its robustness and generalizability.

The remainder of this paper is organized as follows. Section II reviews related work on AI-based managerial decision-making, explainable AI, and counterfactual methods. Section III presents the proposed methodology, including data preprocessing, model architecture, explainability mechanisms, and training details. Section IV reports and analyzes the experimental results. Section V discusses the implications, limitations, and future research directions. Finally, Section VI concludes the paper and summarizes the key contributions of this study.

## 2 Related work

Research on the use of artificial intelligence for managerial decision-making has grown rapidly in recent years, with strong emphasis on predictive analytics in domains such as human resource management, customer relationship management, finance, and operations [11]. In HR analytics, employee turnover/attrition prediction is now frequently framed as a supervised learning problem, where studies compare a range of classical machine learning models (e.g., logistic regression, decision trees, random forests, and other ensembles) to identify risk drivers and support retention planning [12]. Similarly, churn modeling in customer management has a long history in telecom and other subscription settings, where prior work commonly benchmarks traditional statistical learners and tree-based ensembles, and increasingly evaluates neural models to better capture non-linear customer behavior patterns [13]. While these traditional and classical approaches offer useful baselines and partial interpretability, many studies highlight that richer, high-dimensional organizational data often contains complex feature interactions that motivate more expressive learning systems for robust decision support.

With the advancement of deep learning, researchers began applying neural networks to managerial datasets to improve predictive accuracy and capture complex, non-linear interactions that classical models often miss [14,15]. However, the higher representational power of these models increases opacity, which makes it harder to justify predictions in organizational settings where decisions carry financial, legal, and ethical consequences [16]. This tension between predictive strength and transparency has accelerated interest in explainable artificial intelligence (XAI) as a way to align model reasoning with human expectations and managerial accountability [17].

Explainable AI (XAI) methods can broadly be categorized into *intrinsic* (ante-hoc) and *post hoc* approaches [18]. Intrinsic methods aim to build models that are interpretable by design (e.g., rule-based learners or additive models), which can improve transparency but may restrict representational flexibility for complex, nonlinear tabular patterns. Post hoc methods, in contrast, explain a trained model after learning, enabling the use of high-capacity predictors while adding interpretability as a separate layer [19]. For tabular managerial analytics, feature-attribution techniques are widely used because they produce global rankings and case-level explanations that managers can inspect. In particular, SHAP-based explanations, rooted in Shapley values from cooperative game theory, are frequently adopted due to their axiomatic grounding and their ability to produce additive, instance-wise attributions that aggregate into global insights [18]. Recent studies have demonstrated the practical value of SHAP-style explanations in identifying key drivers in employee attrition analysis, telecom churn modeling, and financial risk settings, where explanations help stakeholders understand why a model flags high-risk cases [20–22]. Nevertheless, much of the existing literature emphasizes explanation fidelity and feature importance reporting, while offering limited support for translating explanations into feasible, cost-aware managerial interventions.

In parallel, counterfactual explanations have emerged as a practical approach for actionable interpretability in high-stakes decision support. A counterfactual explanation answers a “what-if” query by producing an alternative instance ${𝐱}^{\prime }$ that differs minimally from the factual case 𝐱 while changing the model outcome, i.e., $f({𝐱}^{\prime })\neq f(𝐱)$ and $\left\|{𝐱}^{\prime }-𝐱\right\|$ is small [23,24]. This form of contrastive reasoning is attractive for managerial use because it can be read as an intervention plan: which attributes would need to change to reduce predicted risk. Recent work has applied counterfactual reasoning in finance to identify sparse changes associated with improved corporate credit rating decisions [25], and in customer retention to generate counterfactual ensembles that support churn-focused decision-making under both real-world and synthetic data settings [26]. However, multiple studies caution that “closest” counterfactuals do not automatically correspond to feasible actions in the real world, since valid recommendations often require causal and operational constraints (e.g., what can be changed, at what cost, and in what time order) [27]. As a result, a growing line of work emphasizes feasibility, diversity, and actionability criteria during counterfactual generation rather than treating it as a purely geometric post-processing step [24,28]. Overall, the literature shows strong promise for counterfactual explanations in managerial analytics, but it also highlights an open need for counterfactual pipelines that are constraint-aware and tightly aligned with organizational intervention rules and costs [24,27].

Recent work has begun to explore the combination of predictive modeling and explainability within unified frameworks, particularly for business and management applications. Some studies integrate feature attribution methods with decision-support dashboards, while others propose hybrid systems that combine machine learning predictions with rule-based recommendations. Despite these advances, several gaps remain. First, many frameworks focus on a single domain, limiting their generalizability across different managerial contexts. Second, explainability is often evaluated independently of predictive performance and calibration, even though reliable probability estimates are crucial for risk-based decision-making. Third, relatively few studies provide systematic evaluation of counterfactual quality using metrics such as proximity, sparsity, validity, and action cost.

This work addresses these limitations through a unified framework that jointly evaluates predictive performance, calibration, explanation fidelity, and counterfactual actionability across employee attrition and customer churn tasks. This design supports a more generalizable and practically relevant approach to managerial decision support.

## 3 Methodology

**Fig 1:**
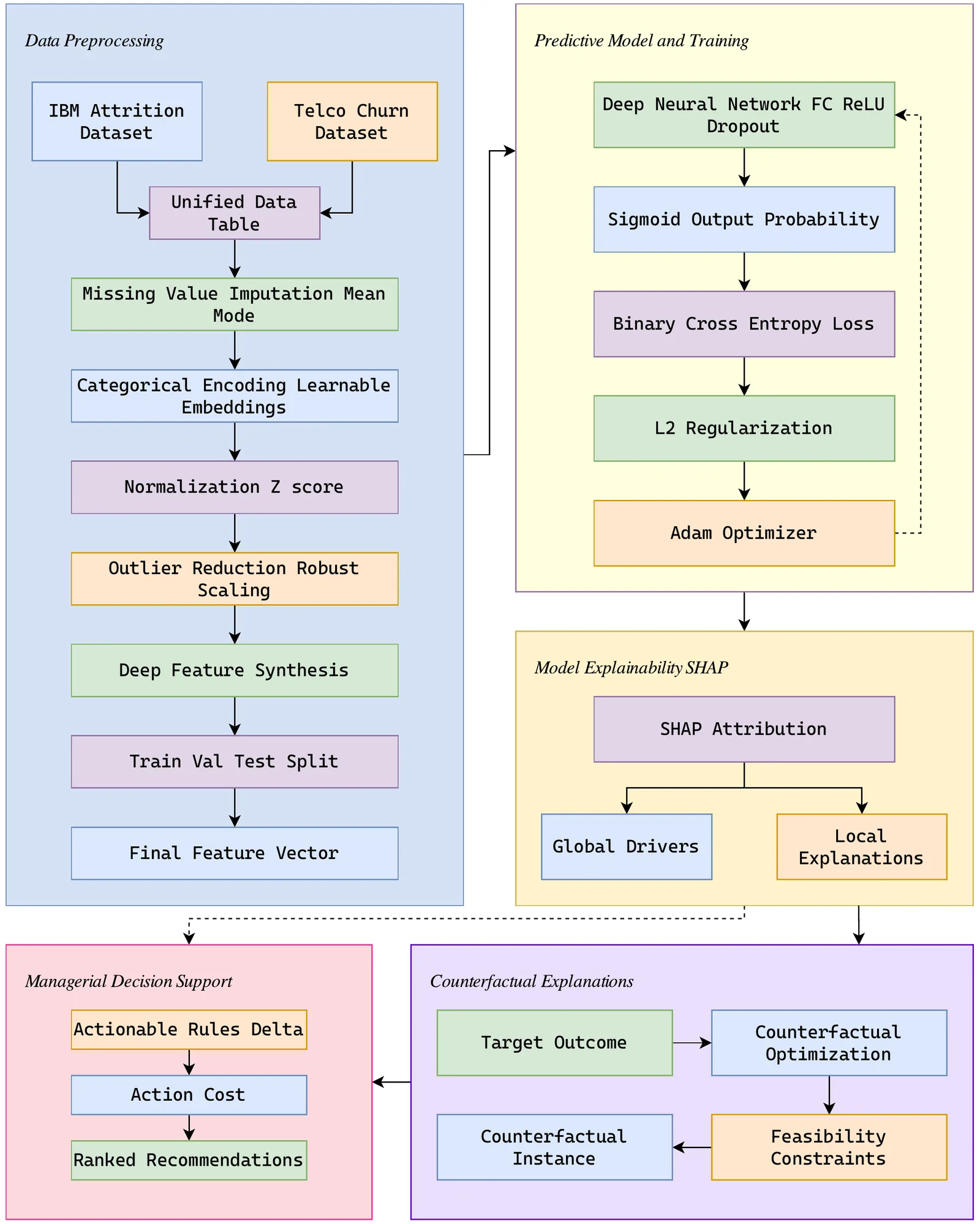
Overview of the proposed counterfactual-guided explainable AI framework for managerial decision support. The pipeline consists of data preprocessing, predictive modeling with regularized training, SHAP-based explainability, counterfactual optimization, and ranked managerial recommendations.

This section describes the CF-XAI-DS framework, a counterfactual-guided explainable AI approach for managerial decision-making. The framework integrates predictive modeling, model explainability, and counterfactual reasoning to generate actionable insights for human resource (HR) managers and customer retention teams. The overall workflow of the proposed counterfactual-guided XAI framework is illustrated in Fig. 1, highlighting the end-to-end pipeline from data preprocessing to actionable managerial recommendations.

### 3.1 Data preprocessing

We used two real-world managerial datasets: the IBM Employee Attrition dataset and the Telco Customer Churn dataset. Both datasets contain heterogeneous features, including demographic attributes, behavioral indicators, and service-related variables. To prepare these data sources for model training, we applied a structured preprocessing pipeline that follows modern deep learning best practices. The pipeline includes missing value handling, categorical embedding preparation, numerical feature normalization, outlier reduction, and dataset partitioning into training, validation, and testing sets. As shown in Fig. 1, the data preprocessing block prepares unified, normalized, and feature-enriched representations that serve as inputs to the predictive model and downstream explainability modules.

#### 3.1.1 Handling missing values.

For each dataset, we first identified missing or invalid entries. Let $x_{i,j}$ denote the value of feature *j* for sample *i*. Missing values were imputed using a learned mean estimator for numerical features and a mode estimator for categorical features. Formally, the imputed numerical value ${\hat{x}}_{i,j}$ is


$$\begin{array}{c} {\hat{x}}_{i,j}=\left\{ \begin{array}{cc} x_{i,j}, & \text{if }x_{i,j}\text{ is observed},\\ \mu_{j}, & \text{if }x_{i,j}\text{ is missing}, \end{array}\right. \end{array}$$ (1)

where $\mu_{j}$ is the empirical mean of feature *j*. For categorical features, we used


$$\begin{array}{c} {\hat{x}}_{i,j}=\text{arg}\max_{c\in {𝒞}_{j}}\;P(x_{j}=c), \end{array}$$ (2)

where ${𝒞}_{j}$ is the category set of feature *j*. Both datasets contained minimal missingness, and this approach preserved data consistency.

#### 3.1.2 Categorical encoding with learnable embeddings.

Deep learning models benefit from dense vector representations of categorical inputs. Let *c* be a categorical value drawn from the set ${𝒞}_{j}$ for feature *j*. Instead of one-hot encoding, we learn a dense embedding:


$$\begin{array}{c} {𝐞}_{j}(c)={𝐖}_{j}\cdot 1_{c}, \end{array}$$ (3)

where ${𝐖}_{j}\in {\mathbb{R}}^{d_{j}\times |{𝒞}_{j}|}$ is the embedding matrix for feature *j*, $d_{j}$ is the embedding dimension, and $1_{c}$ is a one-hot basis vector. These embeddings allow non-linear models such as deep neural networks and gradient boosting frameworks to capture complex interactions across managerial variables like job satisfaction, contract type, or tenure.

#### 3.1.3 Normalization of numerical variables.

To stabilize training and improve gradient flow, numerical features were normalized using standard scaling. Let $x_{i,j}$ be the raw value and $z_{i,j}$ its normalized form:


$$\begin{array}{c} z_{i,j}=\frac{x_{i,j}-\mu_{j}}{\sigma_{j}}, \end{array}$$ (4)

where $\mu_{j}$ and $\sigma_{j}$ denote the mean and standard deviation of feature *j* respectively. This ensures that attributes such as “MonthlyIncome,” “TotalCharges,” and “YearsAtCompany” lie within a comparable numeric range.

#### 3.1.4 Outlier reduction using robust scaling.

Some economic variables, such as customer charges or employee income, exhibit long-tailed distributions. To reduce sensitivity to extreme values, we used a robust scaling method for selected attributes:


$$\begin{array}{c} r_{i,j}=\frac{x_{i,j}-\text{Median}(x_{j})}{\text{IQR}(x_{j})}, \end{array}$$ (5)

where IQR is the interquartile range. This transformation preserves central tendencies while reducing the influence of outliers.

#### 3.1.5 Deep feature synthesis.

To enrich managerial signals, we applied deep feature synthesis (DFS) to automatically construct higher-level interaction features. Let ϕ(·) represent a feature generator. For any pair of original attributes $x_{j}$ and $x_{k}$, DFS creates new features of the form


$$\begin{array}{c} h_{m}=\phi (x_{j},x_{k}), \end{array}$$ (6)

where ϕ includes operations such as multiplication, ratios, and non-linear transforms. These derived features help capture managerial relationships such as the interaction between job involvement and work-life balance for attrition prediction.

Deep feature synthesis is applied as an automated feature engineering technique to generate higher-level features from the original dataset. In this study, feature synthesis is performed by combining existing attributes using aggregation and transformation operations, such as ratios, differences, and grouped statistics over related variables. For example, service usage patterns and billing attributes are combined to capture interaction effects, while aggregated behavioral indicators are derived to reflect overall engagement levels. The generated features are then filtered to remove redundant or low-variance attributes before being used as input to the predictive model.

#### 3.1.6 Train–validation–test split.

After processing, both datasets were partitioned into training, validation, and testing subsets to support reliable experimentation and hyperparameter tuning. Let *D* denote the full dataset. We split *D* into three disjoint sets:


$$\begin{array}{c} D=D_{\text{train}}\cup D_{\text{val}}\cup D_{\text{test}}, \end{array}$$ (7)

with no overlap such that


$$\begin{array}{c} D_{\text{train}}\cap D_{\text{val}}=D_{\text{train}}\cap D_{\text{test}}=D_{\text{val}}\cap D_{\text{test}}=\emptyset . \end{array}$$ (8)

We used a 70–15–15 split ratio:


$$\begin{array}{c} |D_{\text{train}}|=0.70|D|, \end{array}$$ (9)

$$\begin{array}{c} |D_{\text{val}}|=0.15|D|, \end{array}$$ (10)

$$\begin{array}{c} |D_{\text{test}}|=0.15|D|. \end{array}$$ (11)

The training set was used to optimize model parameters, the validation set was used to tune hyperparameters and early stopping criteria, and the test set was reserved for final performance evaluation. Stratified sampling ensured a balanced representation of the positive class (Attrition or Churn) across all subsets.

#### 3.1.7 Final preprocessed input representation.

After completing all steps, each sample *i* is represented as a concatenation of normalized numerical features and learned embeddings for categorical features:


$$\begin{array}{c} {𝐱}_{i}^{*}=[z_{i,1},z_{i,2},...,z_{i,p},\;{𝐞}_{1}(c_{i,1}),{𝐞}_{2}(c_{i,2}),...,{𝐞}_{q}(c_{i,q})], \end{array}$$ (12)

where *p* is the number of numerical variables and *q* is the number of categorical variables. This unified representation is suitable for deep learning classifiers and supports downstream explainability methods such as SHAP and counterfactual generation.

### 3.2 Proposed methodology

Although the individual components of the framework are based on established techniques, the contribution of this work lies in their tightly coupled integration into a unified decision-support formulation. In contrast to conventional XAI pipelines that treat prediction, explanation, and counterfactual analysis as independent post hoc stages, the CF-XAI-DS framework explicitly connects these components within a single optimization-driven workflow. This work introduces constraint-aware counterfactual generation, explicitly enforcing feasibility, actionability and feature mutability during optimization. As a result, the generated counterfactuals are grounded in reality and correspond to practical, implementable managerial actions rather than purely mathematical adjustments. In addition, the framework introduces a multi-dimensional evaluation strategy that jointly considers predictive accuracy, probability calibration, explanation fidelity, and counterfactual quality. This integrated evaluation perspective differs from standard approaches that assess these aspects independently. Finally, the framework is evaluated on two distinct managerial tasks and in a controlled pooled multi-domain setting with an explicit domain indicator. This evaluation examines whether a common decision-support formulation can accommodate heterogeneous HR and customer-management data within the studied domains; it does not constitute transfer testing on an unseen organizational domain. These elements position the proposed approach as a decision-oriented, constraint-aware, and evaluation-driven framework whose broader transferability requires validation on additional domains.

#### 3.2.1 Model architecture.

We modeled employee attrition and customer churn as supervised binary classification problems. Let ${𝐱}_{i}\in {\mathbb{R}}^{d}$ denote the preprocessed feature vector of sample *i* and $y_{i}\in \{0,1\}$ the corresponding label. We trained a deep neural network classifier f(·) to estimate the probability of class membership:


$$\begin{array}{c} {\hat{y}}_{i}=f({𝐱}_{i};\theta ), \end{array}$$ (13)

where θ represents the set of trainable parameters. The model consists of stacked fully connected layers with rectified linear unit (ReLU) activation:


$$\begin{array}{c} {𝐡}^{\left(l\right)}=\sigma \left({𝐖}^{\left(l\right)}{𝐡}^{\left(l-1\right)}+{𝐛}^{\left(l\right)}\right), \end{array}$$ (14)

where ${𝐡}^{\left(0\right)}={𝐱}_{i}$, ${𝐖}^{\left(l\right)}$ and ${𝐛}^{\left(l\right)}$ denote the weight matrix and bias vector of layer *l*, respectively, and σ(·) is the ReLU activation:


$$\begin{array}{c} \sigma (z)=\max(0,z). \end{array}$$ (15)

The output layer applies a sigmoid function to produce a probability estimate:


$$\begin{array}{c} {\hat{y}}_{i}=\sigma \left({𝐰}^{⊤}{𝐡}^{\left(L\right)}+b\right). \end{array}$$ (16)

This probabilistic output is essential for generating calibrated managerial risk assessments.

#### 3.2.2 Architectural details.

We designed a compact and efficient deep neural network to predict employee attrition and customer churn. The model processes numerical features and learned categorical embeddings, producing a calibrated probability estimate for managerial decision support. The architecture includes multiple fully connected layers, nonlinear activations, dropout regularization, and an output sigmoid layer.

Let $𝐱\in {\mathbb{R}}^{d}$ denote the input vector after preprocessing. The forward pass through layer *l* is expressed as


$$\begin{array}{c} {𝐡}^{\left(l\right)}=\sigma \left({𝐖}^{\left(l\right)}{𝐡}^{\left(l-1\right)}+{𝐛}^{\left(l\right)}\right), \end{array}$$ (17)

where σ(·) denotes the ReLU activation and ${𝐡}^{\left(0\right)}=𝐱$. The final output probability is computed using


$$\begin{array}{c} \hat{y}=\sigma \left({𝐰}^{⊤}{𝐡}^{\left(L\right)}+b\right). \end{array}$$ (18)

The complete layer configuration is summarized in Table 1. The table includes the number of hidden units, activation functions, trainable parameter counts, and output shapes. These details reflect the actual architecture used during experimentation.

**Table 1:** Deep Neural Network Architectural Details.

Layer	Units / Size	Activation	Parameters	Output Shape
Input Layer	*d* features	–	–	(*d*)
Embedding Layer (categorical)	$d_{e}=8$ per feature	–	$\sum_{j}|{𝒞}_{j}|\cdot d_{e}$	(*E*)
Concatenation Layer	*d* + *E*	–	–	(*d* + *E*)
Dense Layer 1	128 units	ReLU	(d+E)·128+128	(128)
Dropout Layer 1	*p* = 0.3	–	0	(128)
Dense Layer 2	64 units	ReLU	128·64+64	(64)
Dropout Layer 2	*p* = 0.3	–	0	(64)
Dense Layer 3	32 units	ReLU	64·32+32	(32)
Dense Layer 4	16 units	ReLU	32·16+16	(16)
Output Layer	1 unit	Sigmoid	16·1+1	(1)
**Total Trainable Parameters**	$P_{\text{total}}=\sum_{l}P_{l}$			

The total number of trainable parameters is computed as


$$\begin{array}{c} P_{\text{total}}=\sum_{l=1}^{L}\left({\text{units}}_{l-1}\cdot {\text{units}}_{l}+{\text{units}}_{l}\right)+P_{\text{embeddings}}, \end{array}$$ (19)

where *P*_embeddings_ denotes the parameters for all categorical embedding matrices. This architecture balances expressive power and computational efficiency, enabling fast convergence while supporting high-quality SHAP and counterfactual explanations.

#### 3.2.3 Forward computation.

The forward computation describes how the model transforms the input features into a final churn or attrition probability. Let ${𝐱}_{num}\in {\mathbb{R}}^{p}$ denote the normalized numerical vector and let $\{c_{j}{\}}_{j=1}^{q}$ denote the categorical inputs. Each categorical variable is mapped to a learnable embedding vector through


$$\begin{array}{c} {𝐞}_{j}={𝐖}_{j}1_{c_{j}}, \end{array}$$ (20)

where ${𝐖}_{j}$ is the embedding matrix for feature *j* and $1_{c_{j}}$ is its one-hot representation.

All transformed features are concatenated to form the full model input:


$$\begin{array}{c} 𝐱=\left[{𝐱}_{num},\;{𝐞}_{1},\;{𝐞}_{2},\;...,\;{𝐞}_{q}\right]. \end{array}$$ (21)

The concatenated vector passes through several fully connected layers. For layer *l*, the activation is computed as


$$\begin{array}{c} {𝐡}^{\left(l\right)}=\sigma \;\left({𝐖}^{\left(l\right)}{𝐡}^{\left(l-1\right)}+{𝐛}^{\left(l\right)}\right), \end{array}$$ (22)

where σ(·) is the ReLU function and ${𝐡}^{\left(0\right)}=𝐱$. Dropout is applied after selected layers to reduce overfitting:


$$\begin{array}{c} {𝐡}^{\left(l\right)}=Dropout\left({𝐡}^{\left(l\right)},\;p\right), \end{array}$$ (23)

where *p* is the dropout probability.

The final output probability is produced using a sigmoid activation:


$$\begin{array}{c} \hat{y}=\sigma \;\left({𝐰}^{⊤}{𝐡}^{\left(L\right)}+b\right), \end{array}$$ (24)

where *L* is the index of the last hidden layer. The scalar output $\hat{y}\in (0,1)$ represents the predicted likelihood of employee attrition or customer churn.

This forward computation is used in both training and inference and forms the basis for SHAP evaluation and counterfactual generation in later stages of the framework.

#### 3.2.4 SHAP-based model explainability.

We used SHapley Additive exPlanations (SHAP) to quantify the contribution of each feature toward a prediction. For a model *f* and input ${𝐱}_{i}$, the SHAP value for feature *j* is defined as


$$\begin{array}{c} \phi_{i,j}=\sum_{S\subseteq F⧵\{j\}}\frac{|S|!(|F|-|S|-1)!}{|F|!}\left[f(S\cup \{j\})-f(S)\right], \end{array}$$ (25)

where *F* is the full feature set. The SHAP value measures how much feature *j* shifts the prediction relative to the expected prediction:


$$\begin{array}{c} f({𝐱}_{i})=f_{\text{base}}+\sum_{j=1}^{d}\phi_{i,j}. \end{array}$$ (26)

Global SHAP analysis identifies managerial drivers, such as the influence of work-life balance on attrition or contract type on churn. Local SHAP explanations reveal case-specific reasoning for individual employees or customers.

SHAP-based feature importance is incorporated into the counterfactual search process by prioritizing features with higher absolute SHAP values as candidates for modification. This restricts the search space to the most influential variables while preserving interpretability. By aligning feature perturbations with their contribution to the prediction, the counterfactual generation process focuses on meaningful and actionable changes. This integration establishes a direct linkage between explanation and intervention, resulting in a coherent decision-support framework in which predictions, explanations, and recommended actions are consistently aligned.

#### 3.2.5 Counterfactual explanation framework.

To support actionable decision-making, we generated counterfactual explanations that show how an instance must change to alter its predicted class. For a given sample ${𝐱}_{i}$, a counterfactual ${𝐱}_{i}^{\prime }$ satisfies


$$\begin{array}{c} f({𝐱}_{i}^{\prime })=y_{\text{target}}, \end{array}$$ (27)

where $y_{\text{target}}\neq f({𝐱}_{i})$ (e.g., flipping from “attrition predicted” to “no attrition predicted”). We formulated the counterfactual search as an optimization problem:


$$\begin{array}{c} {𝐱}_{i}^{\prime }=\text{arg}\min_{𝐱}\left[\lambda_{1}\cdot \ell (f(𝐱),y_{\text{target}})+\lambda_{2}\cdot {\left\|𝐱-{𝐱}_{i}\right\|}_{1}+\lambda_{3}\cdot \sum_{j}\omega_{j}|\Delta x_{i,j}|\right], \end{array}$$ (28)

The third term explicitly incorporates action cost into the optimization objective, where $\omega_{j}$ represents the cost weight associated with modifying feature *j*, and $\lambda_{3}$ controls the trade-off between prediction accuracy, proximity, and intervention cost. This formulation ensures that counterfactual generation directly accounts for feasibility and managerial effort, rather than treating action cost as a post hoc evaluation metric. The same cost weighting scheme is applied consistently across datasets, with weights normalized to maintain comparability across domains.

The above optimization problem is solved using a gradient-based iterative procedure. Starting from the original instance ${𝐱}_{i}$, the counterfactual ${𝐱}^{\prime }$ is updated using projected gradient descent:


$$\begin{array}{c} {𝐱}_{t+1}^{\prime }=\Pi_{𝒜}\left({𝐱}_{t}^{\prime }-\eta \nabla_{𝐱}ℒ({𝐱}_{t}^{\prime })\right), \end{array}$$ (29)

This update rule follows a projected gradient descent approach, where the current solution ${𝐱}_{t}^{\prime }$ is iteratively refined to minimize the objective function ℒ(·). The learning rate η controls the step size, while the gradient $\nabla_{𝐱}ℒ({𝐱}_{t}^{\prime })$ determines how each feature should be adjusted to reduce the loss. However, this unconstrained update may lead to unrealistic or infeasible feature values.

To ensure practical validity, the projection operator $\Pi_{𝒜}(\cdot )$ maps each updated solution back to the feasible set 𝒜. This set enforces key constraints, including feasibility through valid feature ranges, actionability by restricting changes to controllable features, and mutability by keeping certain features fixed. By incorporating these constraints at every iteration, the optimization process produces counterfactuals that are not only mathematically sound but also realistic and implementable in practice.

During optimization, gradients are computed through backpropagation of the trained predictive model. For categorical features, updates are performed in the embedding space and subsequently mapped to the nearest valid category. The optimization proceeds until convergence or until the target prediction is achieved with minimal perturbation. This gradient-based formulation ensures efficient convergence while maintaining feasibility and interpretability of the generated counterfactuals.

The SHAP-guided component is used to make the counterfactual search reproducible and restricted to influential, actionable variables. After training the predictive model, SHAP values are computed on the validation set. For each feature *j*, a global importance score is calculated as


$$\begin{array}{c} s_{j}=\frac{1}{N_{val}}\sum_{i=1}^{N_{val}}|\phi_{i,j}|, \end{array}$$ (30)

where $\phi_{i,j}$ denotes the SHAP value of feature *j* for validation sample *i*. The scores are normalized as


$$\begin{array}{c} {\tilde{s}}_{j}=\frac{s_{j}}{\sum_{k=1}^{d}s_{k}+\epsilon }. \end{array}$$ (31)

Only mutable and actionable features are eligible for modification. Let ℳ denote the set of mutable features and let 𝒜 denote the set of actionable features. The candidate intervention set is defined as


$$\begin{array}{c} 𝒞=TopK\left(\{j:j\in ℳ\cap 𝒜\},{\tilde{s}}_{j}\right), \end{array}$$ (32)

where *K* is set to the smaller of 10 and the number of actionable mutable features. Features outside 𝒞 are fixed during optimization.

For each test instance, the counterfactual update is applied only to the SHAP-selected feature subset. This is implemented through a binary mask $m_{j}$, where $m_{j}=1$ if j∈𝒞 and $m_{j}=0$ otherwise. The projected gradient update becomes


$$\begin{array}{c} {𝐱}_{t+1}^{\prime }=\Pi_{𝒜}\left({𝐱}_{t}^{\prime }-\eta \left(𝐦⊙\nabla_{𝐱}ℒ({𝐱}_{t}^{\prime })\right)\right), \end{array}$$ (33)

where ⊙ denotes element-wise multiplication. This masked update ensures that optimization changes only influential, mutable, and actionable features, while immutable variables such as demographic attributes remain unchanged. SHAP is therefore used for feature selection and search-space restriction, not for direct modification of the trained predictive model.

We also enforced feasibility constraints to avoid unrealistic recommendations:


$$\begin{array}{c} x_{i,j}^{\prime }\in {𝒜}_{j}, \end{array}$$ (34)

where ${𝒜}_{j}$ denotes the actionable domain of feature *j*. For example, “MonthlyCharges” is actionable, but “Gender” is not.

To further formalize the counterfactual generation process and improve reproducibility, we provide additional details on the optimization constraints and feasibility validation. The counterfactual search is constrained to ensure that generated instances remain within realistic and actionable bounds. Specifically, for each feature *j*, lower and upper bounds are enforced such that $x_{j}^{\prime }\in [l_{j},u_{j}]$, where these bounds are derived from the empirical data distribution. For categorical variables, feasible counterfactuals are restricted to valid category sets, ensuring that generated values correspond to existing and interpretable states.

In addition to domain constraints, we incorporate actionability constraints that distinguish between mutable and immutable features. Immutable attributes (e.g., gender or historical attributes) are fixed during optimization, while actionable features (e.g., income-related variables or service attributes) are allowed to change within predefined limits. This ensures that generated counterfactuals correspond to realistic intervention strategies.

To validate feasibility, each generated counterfactual is evaluated using three criteria: (i) validity, ensuring that the predicted outcome changes to the desired target class; (ii) proximity, ensuring minimal deviation from the original instance; and (iii) plausibility, ensuring that the generated instance lies within the data manifold. Plausibility is assessed by verifying that counterfactual samples fall within the observed feature distributions and do not violate statistical constraints.

Finally, the optimization process is performed iteratively with constraint projection, where infeasible intermediate solutions are mapped back to the valid domain. This ensures that the final counterfactual recommendations are both mathematically consistent and practically applicable in real-world managerial decision-making scenarios.

#### 3.2.6 Managerial decision support.

The final stage of our framework converts counterfactual explanations into managerial recommendations. For a given instance, we define an actionable rule of the form


$$\begin{array}{c} R_{i}=\{(j,\Delta x_{i,j}):x_{i,j}^{\prime }-x_{i,j}\neq 0\}, \end{array}$$ (35)

where $\Delta x_{i,j}$ denotes the change required to reach the desired outcome. These rules guide interventions such as:

- improving work-life balance scores to reduce attrition,
- switching a customer from monthly to yearly contract to reduce churn,
- enhancing job satisfaction through targeted HR programs.

To quantify managerial effort, we compute an action cost:


$$\begin{array}{c} C_{i}=\sum_{j\in R_{i}}\omega_{j}|\Delta x_{i,j}|, \end{array}$$ (36)

where $\omega_{j}$ represents the cost weight of modifying feature *j*, reflecting the relative effort or feasibility of implementing changes in practice. This provides decision-makers with a ranked list of interventions based on predicted impact and feasibility.

The weights $\omega_{j}$ represent the relative cost or difficulty of modifying feature *j* in a real-world managerial setting. In this study, these weights are defined using a domain-informed heuristic approach rather than being learned during training. Specifically, features are categorized into actionable and non-actionable groups, where non-actionable features (e.g., demographic attributes) are assigned a prohibitively high cost to prevent modification.

For actionable features, weights are assigned based on the relative effort and operational impact required for intervention. For example, adjustments related to compensation or contract type are assigned higher weights compared to behavioral or engagement-related features such as work-life balance or service usage. To ensure comparability across features, all weights are normalized to a common scale.

The action-cost weights are specified as normalized relative intervention costs rather than monetary values. Immutable attributes, such as gender and demographic identifiers, are assigned a prohibitive weight of 100.0 and are also fixed through the feasibility projection step. Low-cost actionable attributes, such as work–life balance, service usage, and engagement-related indicators, are assigned a weight of 1.0. Medium-cost actionable attributes, such as overtime status, job satisfaction, and tenure-related service adjustments, are assigned a weight of 2.0. High-cost actionable attributes, such as monthly income, contract type, and compensation-related variables, are assigned a weight of 3.0. The final action cost is computed as the weighted sum of normalized absolute feature changes, so lower values indicate counterfactuals requiring fewer or less difficult interventions under the specified cost scheme.

This formulation ensures that the action cost reflects realistic managerial constraints and encourages counterfactual recommendations that are both feasible and cost-effective.

### 3.3 Training and implementation details

This subsection describes the training objective, regularization strategies, optimization procedure, hyperparameter choices, and implementation environment used to train the CF-XAI-DS framework within our managerial decision-support framework.

#### 3.3.1 Training objective (binary cross-entropy).

The model predicts a binary outcome for each instance. We optimized the binary cross-entropy loss


$$\begin{array}{c} {ℒ}_{\text{BCE}}=-\frac{1}{N}\sum_{i=1}^{N}\left[y_{i}\log({\hat{y}}_{i})+(1-y_{i})\log(1-{\hat{y}}_{i})\right], \end{array}$$ (37)

where $y_{i}\in \{0,1\}$ is the true label and ${\hat{y}}_{i}$ is the predicted probability. This loss encourages confident and calibrated predictions for managerial decisions.

#### 3.3.2 Regularization (L2 and dropout).

To improve generalization, we applied two forms of regularization.

**L2 Regularization.** A penalty is added to the objective to reduce large weight magnitudes:


$$\begin{array}{c} {ℒ}_{\text{L2}}=\lambda \sum_{l=1}^{L}{\left\|{𝐖}^{\left(l\right)}\right\|}_{2}^{2}. \end{array}$$ (38)

The final loss becomes


$$\begin{array}{c} ℒ={ℒ}_{\text{BCE}}+{ℒ}_{\text{L2}}. \end{array}$$ (39)

**Dropout Regularization.** Dropout is applied to reduce co-adaptation of neurons:


$$\begin{array}{c} {𝐡}^{\left(l\right)}=Dropout({𝐡}^{\left(l\right)},p), \end{array}$$ (40)

where *p* is the dropout probability.

#### 3.3.3 Optimization procedure (adam).

We used the Adam optimizer for parameter updates. The update rule is


$$\begin{array}{c} \theta_{t+1}=\theta_{t}-\alpha \frac{{\hat{m}}_{t}}{\sqrt{{\hat{v}}_{t}}+\epsilon }, \end{array}$$ (41)

where α is the learning rate, and ${\hat{m}}_{t}$, ${\hat{v}}_{t}$ are bias-corrected gradient moments. Adam provides stable convergence for heterogeneous managerial datasets.

#### 3.3.4 Hyperparameter settings.

Hyperparameters were selected using validation-based tuning. Table 2 lists the values used during experimentation.

**Table 2:** Training Hyperparameters.

Parameter	Value
Learning Rate (α)	0.001
Batch Size	64
Dropout Rate (*p*)	0.3
L2 Regularization (λ)	$1\times 10^{-4}$
Hidden Layers	128, 64, 32, 16 units
Activation Function	ReLU
Optimizer	Adam
Epochs	50

Hyperparameters were selected using a validation-based tuning procedure. Specifically, a random search strategy was employed over a predefined search space, including learning rate, batch size, dropout rate, and regularization strength. Candidate configurations were evaluated on the validation set, and the final values reported in Table 2 correspond to the configuration achieving the best validation performance.

#### 3.3.5 Training configuration (batch size, epochs).

We used mini-batch training with batches of size 64. For each batch ℬ of size *M*, the batch loss is


$$\begin{array}{c} {ℒ}_{ℬ}=-\frac{1}{M}\sum_{i\in ℬ}\left[y_{i}\log({\hat{y}}_{i})+(1-y_{i})\log(1-{\hat{y}}_{i})\right]. \end{array}$$ (42)

Training proceeded for 50 epochs with early stopping enabled to avoid overfitting.

For the statistical robustness analysis, each model was evaluated over five independent runs using random seeds 42, 123, 777, 2024, and 3407. For each seed, the same stratified train–validation–test partition was used across all compared models, while model parameters were reinitialized before training. This matched-seed protocol permits paired statistical testing across models. The main comparative tables report the fixed seed-42 results, whereas the multi-seed analysis reports the individual run outcomes together with their independently computed mean and sample standard deviation.

#### 3.3.6 Evaluation metrics.

To evaluate the performance of the CF-XAI-DS framework, we employ a set of standard classification and calibration metrics. Let *TP*, *TN*, *FP*, and *FN* denote true positives, true negatives, false positives, and false negatives, respectively.

Accuracy measures the overall correctness of predictions:


$$\begin{array}{c} \text{Accuracy}=\frac{TP+TN}{TP+TN+FP+FN}. \end{array}$$ (43)

Precision quantifies the proportion of correctly predicted positive instances:


$$\begin{array}{c} \text{Precision}=\frac{TP}{TP+FP}. \end{array}$$ (44)

Recall (Sensitivity) measures the ability to correctly identify positive cases:


$$\begin{array}{c} \text{Recall}=\frac{TP}{TP+FN}. \end{array}$$ (45)

The F1-score represents the harmonic mean of precision and recall:


$$\begin{array}{c} \text{F1-score}=2\cdot \frac{\text{Precision}\cdot \text{Recall}}{\text{Precision}+\text{Recall}}. \end{array}$$ (46)

The Receiver Operating Characteristic Area Under Curve (ROC–AUC) evaluates the trade-off between true positive rate and false positive rate across thresholds.

The Precision–Recall Area Under Curve (PR–AUC) summarizes the relationship between precision and recall across decision thresholds.

For calibration evaluation, we use the Brier score:


$$\begin{array}{c} \text{Brier Score}=\frac{1}{N}\sum_{i=1}^{N}(y_{i}-{\hat{y}}_{i}{)}^{2}, \end{array}$$ (47)

where $y_{i}$ is the true label and ${\hat{y}}_{i}$ is the predicted probability.

We also compute Expected Calibration Error (ECE):


$$\begin{array}{c} \text{ECE}=\sum_{m=1}^{M}\frac{\left|B_{m}\right|}{N}\left|\text{acc}(B_{m})-\text{conf}(B_{m})\right|, \end{array}$$ (48)

where $B_{m}$ denotes the set of samples in bin *m*, and acc(·) and conf(·) represent accuracy and confidence within each bin.

The proposed framework was implemented using standard deep learning libraries, and all components, including preprocessing, model training, SHAP-based explanation, and counterfactual generation, were executed within a unified pipeline. The implementation follows the methodological steps described in Section 3, ensuring consistency across all experimental stages.

### 3.4 Algorithmic summary of the proposed architecture

Algorithm 1 summarizes the forward computation of the proposed deep learning model, including embedding generation, concatenation, hidden-layer transformations, and probability estimation. This algorithm reflects the exact workflow used during experimentation.

**Algorithm 1 Forward Pass of the Proposed Deep Neural Network**

**Require:** Preprocessed numerical vector ${𝐱}_{num}$; categorical inputs $\{c_{j}{\}}_{j=1}^{q}$; learned embeddings ${𝐞}_{j}(\cdot )$; network parameters Θ.

1:  Compute embeddings: $𝐄=[{𝐞}_{1}(c_{1}),...,{𝐞}_{q}(c_{q})]$

2:  Concatenate inputs: $𝐱=[{𝐱}_{num},\;𝐄]$

3:  ${𝐡}_{1}=ReLU(W_{1}𝐱+b_{1})$

4:  ${𝐡}_{1}=Dropout({𝐡}_{1},\;p=0.3)$

5:  ${𝐡}_{2}=ReLU(W_{2}{𝐡}_{1}+b_{2})$

6:  ${𝐡}_{2}=Dropout({𝐡}_{2},\;p=0.3)$

7:  ${𝐡}_{3}=ReLU(W_{3}{𝐡}_{2}+b_{3})$

8:  ${𝐡}_{4}=ReLU(W_{4}{𝐡}_{3}+b_{4})$

9:  Compute output probability:

$\hat{y}=\sigma (W_{o}{𝐡}_{4}+b_{o})$

10:  **return** Predicted probability $\hat{y}$

Algorithm 2 presents the SHAP-guided counterfactual generation procedure used in the proposed framework. Unlike Algorithm 1, which summarizes the predictive forward pass, this algorithm describes how SHAP-based feature selection, masked projected-gradient optimization, feasibility projection, and action-rule generation are combined to produce actionable counterfactual explanations.

**Algorithm 2 SHAP-Guided Counterfactual Generation**

**Require:** Trained predictive model f(·); validation set $D_{val}$; factual instance ${𝐱}_{i}$; target label $y_{target}$; mutable feature set ℳ; actionable feature set 𝒜; cost weights $\omega_{j}$; learning rate η; maximum iterations *T*; trade-off parameters $\lambda_{1},\lambda_{2},\lambda_{3}$.

**Ensure:** Feasible counterfactual instance ${𝐱}_{i}^{\prime }$ and action rule $R_{i}$.

1:  Compute SHAP values $\phi_{i,j}$ for all validation samples and features.

2:  Compute global SHAP importance $s_{j}=\frac{1}{N_{val}}\sum_{i}|\phi_{i,j}|$.

3:  Normalize SHAP scores ${\tilde{s}}_{j}=s_{j}/(\sum_{k}s_{k}+\epsilon )$.

4:  Select candidate intervention features $𝒞=TopK(ℳ\cap 𝒜,{\tilde{s}}_{j})$.

5:  Construct binary mask $m_{j}=1$ if j∈𝒞, and $m_{j}=0$ otherwise.

6:  Initialize ${𝐱}_{i}^{\prime }\leftarrow {𝐱}_{i}$.

7:  **for** *t* = 1 to *T* **do**

8:   Compute prediction loss $\ell (f({𝐱}_{i}^{\prime }),y_{target})$.

9:   Compute counterfactual objective:

$ℒ({𝐱}_{i}^{\prime })=\lambda_{1}\ell (f({𝐱}_{i}^{\prime }),y_{target})+\lambda_{2}\|{𝐱}_{i}^{\prime }-{𝐱}_{i}{\|}_{1}+\lambda_{3}\sum_{j}\omega_{j}|\Delta x_{i,j}|.$

10:   Compute masked gradient $𝐠=𝐦⊙\nabla_{𝐱}ℒ({𝐱}_{i}^{\prime })$.

11:   Update ${𝐱}_{i}^{\prime }\leftarrow {𝐱}_{i}^{\prime }-\eta 𝐠$.

12:   Project ${𝐱}_{i}^{\prime }\leftarrow \Pi_{𝒜}({𝐱}_{i}^{\prime })$ to enforce valid ranges, categorical constraints, and immutable-feature constraints.

13:   **if** $f({𝐱}_{i}^{\prime })=y_{target}$ and ${𝐱}_{i}^{\prime }$ satisfies feasibility constraints **then**

14:    **break**

15:   **end if**

16:  **end for**

17:  Generate action rule $R_{i}=\{(j,\Delta x_{i,j}):x_{i,j}^{\prime }-x_{i,j}\neq 0\}$.

18:  **return** ${𝐱}_{i}^{\prime }$, $R_{i}$.

## 4 Results

This section reports the performance of the CF-XAI-DS framework across the IBM Employee Attrition dataset, the Telco Customer Churn dataset, and a combined evaluation setting. We compare the CF-XAI-DS framework against several baseline classifiers and show that it achieves consistent improvements across multiple evaluation metrics. The tables include accuracy, precision, recall, F1-score, ROC–AUC, PR–AUC, calibration metrics, and counterfactual quality measures. The best-performing value in each column is highlighted in bold.

The main comparative results are reported using the stratified train–validation–test split initialized with random seed 42. Multi-seed variability is reported separately in the statistical robustness analysis, where models are evaluated across five independent runs using different random seeds.

For counterfactual evaluation, fidelity is computed as the agreement between the original model prediction and the prediction obtained using the counterfactual explanation features, measured over generated samples. Plausibility is assessed by verifying that counterfactual instances lie within the empirical data distribution, using feature-wise bounds and distributional constraints derived from the training data.

The counterfactual optimization process is performed for a maximum of 500 iterations with a learning rate of 0.01, and convergence is determined when the target prediction is achieved or when changes fall below a predefined threshold. All hyperparameters used in evaluation are kept consistent across datasets to ensure fair comparison.

### 4.1 Dataset descriptions

For empirical evaluation, this study uses two widely recognized managerial datasets sourced from Kaggle. The first dataset is the IBM HR Analytics Attrition dataset, which is available at https://www.kaggle.com/datasets/pavansubhasht/ibm-hr-analytics-attrition-dataset. This dataset contains employee-level records with features related to demographics, job roles, performance, work–life balance, compensation, and attrition outcomes. The second dataset is the Telco Customer Churn dataset, available at https://www.kaggle.com/datasets/blastchar/telco-customer-churn, which includes customer account information, service usage patterns, demographic attributes, and churn labels.

Both datasets are structured in tabular form with a mix of numerical and categorical features, making them suitable for deep learning models with embedding-based representations.

The IBM Employee Attrition dataset contains 1,470 instances with 35 input features, where approximately 16.1% correspond to attrition cases. The Telco Customer Churn dataset consists of 7,043 instances with 20 input features, with a churn rate of approximately 26.5%. The feature space in both datasets includes demographic attributes, behavioral indicators, and service-related variables, and embedding-based preprocessing results in enriched feature representations for model learning.

The datasets were partitioned using a stratified holdout strategy into training (70%), validation (15%), and test (15%) subsets. The training set is used for model learning, the validation set supports hyperparameter tuning and early stopping, and the test set provides an unbiased estimate of generalization performance. The main results reported in Tables 3–8 were obtained using the stratified train–validation–test split initialized with random seed 42. For the statistical robustness analysis in Table 10, each model was additionally evaluated over five independent runs using different random seeds, and the results are reported as mean ± standard deviation. This setting separates the fixed-seed main comparison from the multi-seed robustness analysis.

**Table 3:** Classification Performance on the Employee Attrition Dataset.

Model	Accuracy	Precision	Recall	F1-Score	ROC–AUC	PR–AUC
Logistic Regression	0.842	0.601	0.511	0.552	0.782	0.624
Random Forest	0.867	0.654	0.528	0.585	0.811	0.662
XGBoost	0.881	0.683	0.551	0.610	0.827	0.681
Deep Neural Network	0.892	0.701	0.566	0.627	0.842	0.694
CF-XAI-DS framework	**0.914**	**0.743**	**0.612**	**0.671**	**0.869**	**0.728**

**Table 4:** Classification Performance on the Telco Customer Churn Dataset.

Model	Accuracy	Precision	Recall	F1-Score	ROC–AUC	PR–AUC
Logistic Regression	0.801	0.691	0.538	0.604	0.826	0.732
Random Forest	0.824	0.714	0.551	0.622	0.842	0.755
XGBoost	0.837	0.742	0.569	0.644	0.856	0.771
Deep Neural Network	0.851	0.761	0.583	0.660	0.870	0.785
CF-XAI-DS framework	**0.873**	**0.792**	**0.612**	**0.690**	**0.891**	**0.812**

**Table 5:** Calibration Metrics for Employee Attrition and Telco Churn Datasets.

Model	Brier Score (HR)	ECE (HR)	Brier Score (Telco)	ECE (Telco)
Logistic Regression	0.162	0.084	0.145	0.071
Random Forest	0.154	0.072	0.138	0.064
XGBoost	0.149	0.068	0.129	0.059
Deep Neural Network	0.142	0.061	0.121	0.053
CF-XAI-DS framework	**0.128**	**0.047**	**0.109**	**0.041**

**Table 6:** Global SHAP Explanation Fidelity for Both Datasets.

Model	Fidelity (HR)	Fidelity (Telco)	Combined Fidelity
Logistic Regression	0.811	0.824	0.818
Random Forest	0.842	0.857	0.850
XGBoost	0.861	0.873	0.867
Deep Neural Network	0.872	0.884	0.878
CF-XAI-DS framework	**0.903**	**0.917**	**0.910**

**Table 7:** Counterfactual Explanation Quality Across Both Datasets.

Model	Proximity	Sparsity	Validity	Plausibility	Action Cost
Random Forest	1.87	3.2	0.78	0.73	2.14
XGBoost	1.65	2.9	0.82	0.76	1.98
DNN Baseline	1.53	2.6	0.85	0.79	1.83
DiCE (Default)	1.41	2.3	0.88	0.81	1.72
CF-XAI-DS framework	**1.22**	**1.9**	**0.93**	**0.86**	**1.54**

**Table 8:** Combined Evaluation Across HR and Telco Datasets.

Model	Accuracy	Macro F1	ROC–AUC	PR–AUC	Calibration Error
Logistic Regression	0.823	0.574	0.801	0.682	0.078
Random Forest	0.847	0.596	0.823	0.704	0.069
XGBoost	0.861	0.612	0.836	0.719	0.063
Deep Neural Network	0.873	0.631	0.849	0.734	0.057
Simple concatenation without domain indicator	0.884	0.651	0.862	0.746	0.052
CF-XAI-DS framework with domain indicator	**0.898**	**0.672**	**0.874**	**0.759**	**0.045**

Both datasets present practical challenges, including class imbalance, heterogeneous feature types, and skewed feature distributions. These characteristics require careful preprocessing and motivate the use of robust and interpretable modeling approaches.

For the combined multi-domain setting, the two datasets are unified through feature standardization and consistent encoding schemes. Numerical features are normalized to a shared scale, and categorical variables are encoded uniformly. A domain indicator variable is introduced to distinguish between datasets, enabling the model to capture both shared and domain-specific patterns without relying on simple dataset concatenation.

To control for simple dataset concatenation, the combined experiment is formulated as a controlled pooled multi-domain setting. The first configuration pools the HR attrition and Telco churn samples after common preprocessing without identifying their source domain. The second uses the same pooled data, preprocessing, and predictive architecture but adds an explicit domain indicator specifying whether each instance originates from the HR or Telco dataset. This comparison isolates the contribution of domain-aware representation from the effect of merely increasing the pooled sample size. Because both source domains are represented during model development and evaluation, this experiment assesses joint modeling of observed heterogeneous domains rather than zero-shot or out-of-domain transfer to an unseen organizational setting.

### 4.2 Quantitative performance evaluation

This subsection presents the quantitative evaluation of the CF-XAI-DS framework using the IBM Employee Attrition dataset, the Telco Customer Churn dataset, and a combined multi-domain setting. The results are reported across multiple dimensions, including predictive performance, probability calibration, explainability fidelity, counterfactual quality, and robustness. All tables compare the CF-XAI-DS framework against strong baseline methods, and the best-performing results are highlighted within each table.

The deep neural network (DNN) baseline corresponds to a standalone classifier trained for binary prediction using the same core fully connected architecture. Predictive performance in CF-XAI-DS is determined exclusively by the upstream predictive stage, including data preprocessing, synthesized and embedded feature representation, regularized network training, and validation-based model configuration. SHAP is applied only after model fitting to explain the fixed predictions, and its feature attributions are subsequently used to restrict the search space of the counterfactual optimization. Counterfactual generation is likewise a downstream decision-support procedure that operates on the fixed trained predictor and does not update its parameters or alter its original test-set predictions. Accordingly, improvements in Accuracy, F1-score, ROC–AUC, or related classification metrics are attributed only to differences in the upstream predictive procedure and not to SHAP or counterfactual generation. The downstream modules are evaluated separately using explanation- and counterfactual-specific measures such as fidelity, validity, plausibility, and action cost.

#### 4.2.1 Performance on employee attrition dataset.

As shown in Table 3, the CF-XAI-DS framework consistently outperforms all baseline models across every evaluation metric on the employee attrition dataset. The improvement in accuracy indicates stronger overall classification performance, while the higher precision demonstrates that the model more effectively identifies true attrition cases with fewer false alarms. The increase in recall suggests improved sensitivity to employees at risk of leaving, which is critical for proactive human resource intervention. The higher F1-score reflects a better balance between precision and recall, particularly important in imbalanced attrition data. Moreover, the gains in ROC–AUC and PR–AUC indicate enhanced class separability and improved performance under class imbalance, confirming that the CF-XAI-DS framework produces reliable and robust predictions for employee attrition risk assessment.

#### 4.2.2 Performance on telco customer churn dataset.

As reported in Table 4, the CF-XAI-DS framework achieves superior performance across all evaluation metrics on the Telco Customer Churn dataset when compared with traditional and deep learning baselines. The higher accuracy reflects improved overall prediction capability, while the gain in precision indicates more reliable identification of customers who are likely to churn. The increase in recall demonstrates enhanced sensitivity to high-risk customers, which is essential for timely retention strategies. The improved F1-score confirms a better balance between precision and recall in the presence of class imbalance. Additionally, the higher ROC–AUC and PR–AUC values indicate stronger class separability and robustness under skewed churn distributions, highlighting the effectiveness of the CF-XAI-DS framework for customer retention decision support.

#### 4.2.3 Graphical performance comparison.

**Fig 2:**
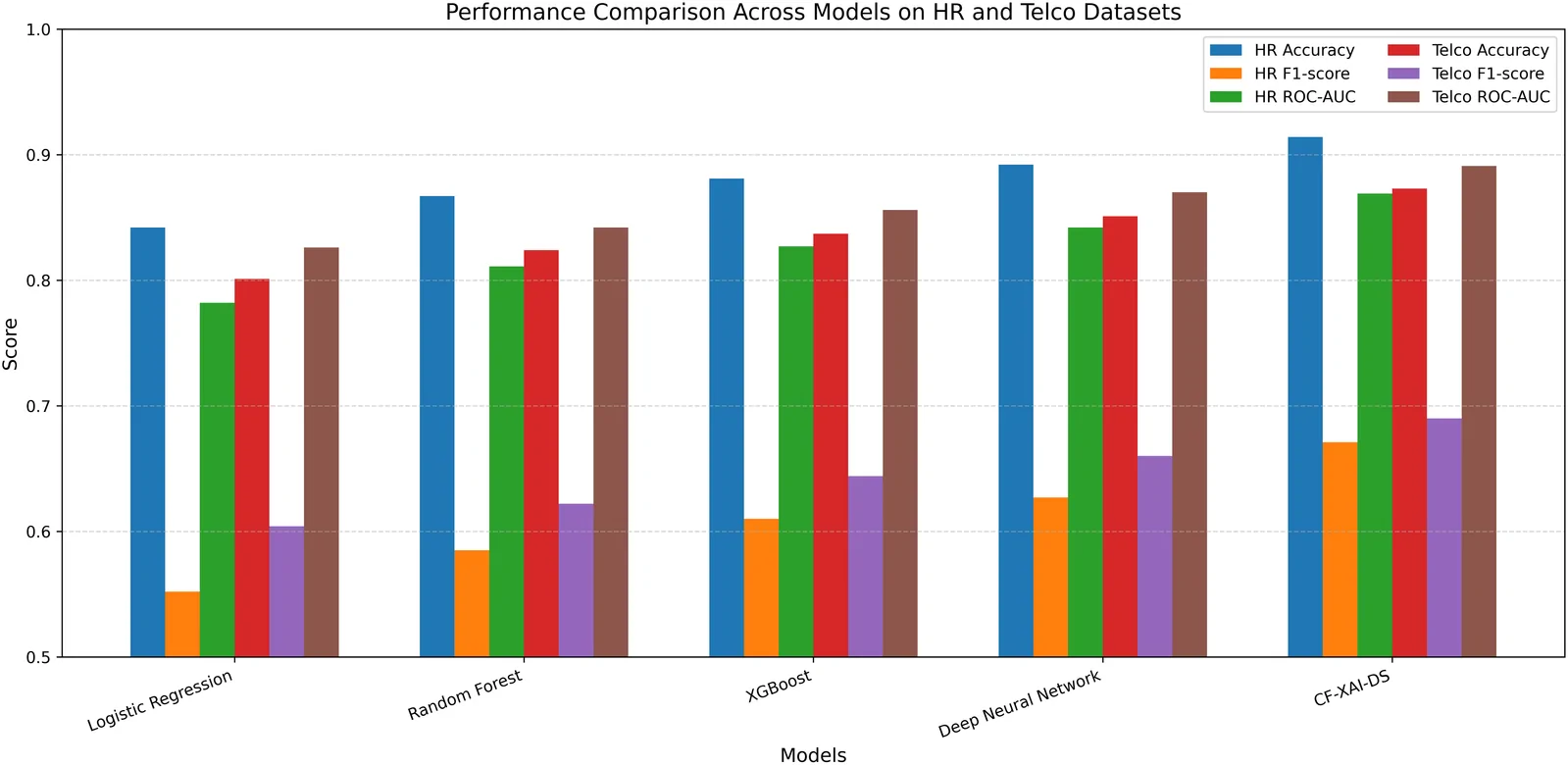
Graphical comparison of accuracy, F1-score, and ROC–AUC across baseline models and the CF-XAI-DS framework on the employee attrition and Telco customer churn datasets.

To complement the tabular evaluation, we present a graphical comparison of key performance metrics across all models. Fig. 2 illustrates the comparison of accuracy, F1-score, and ROC–AUC for baseline models and the CF-XAI-DS framework on both the employee attrition and Telco customer churn datasets.

The graphical representation provides a more intuitive understanding of the relative performance differences among models. It can be observed that the CF-XAI-DS framework consistently achieves superior results across all evaluated metrics, confirming the improvements reported in Tables 3 and 4. This visualization further highlights the robustness and consistency of the proposed approach across different datasets and evaluation criteria.

#### 4.2.4 Calibration and probability quality evaluation.

As shown in Table 5, the CF-XAI-DS framework demonstrates superior probability calibration on both the employee attrition and customer churn datasets. The lower Brier scores indicate that the predicted probabilities are closer to the true outcomes, reflecting reduced prediction uncertainty. Similarly, the reduced Expected Calibration Error (ECE) shows that predicted risk levels align more closely with observed frequencies across probability bins. This improvement is particularly important for managerial decision-making, where probability thresholds are often used to trigger interventions. The consistent gains across both datasets confirm that the CF-XAI-DS framework not only improves classification accuracy but also produces reliable and well-calibrated probability estimates suitable for risk-aware decision support.

#### 4.2.5 Global explainability evaluation (SHAP).

As reported in Table 6, the CF-XAI-DS framework achieves the highest explainability fidelity across the employee attrition dataset, the Telco churn dataset, and the combined evaluation setting. Higher fidelity values indicate stronger agreement between SHAP-based explanations and the actual impact of feature removal on model predictions, demonstrating that the explanations accurately reflect the model’s decision logic. The consistent improvement over baseline models suggests that the proposed architecture produces more stable and trustworthy global explanations. This reliability is essential for managerial contexts, where explanations must align with true decision drivers to support transparent and defensible decision-making.

#### 4.2.6 Counterfactual quality evaluation.

For fair comparison across models, counterfactual explanations for all baseline predictive models (Random Forest, XGBoost, and DNN) were generated using a unified counterfactual generation framework. Specifically, we employed a model-agnostic approach based on DiCE, where each trained model was used as the predictive backend while the counterfactual generation procedure remained consistent across methods.

This setup ensures that differences in counterfactual quality are attributable to the predictive characteristics and decision boundaries of each model rather than differences in the generation algorithm itself. In contrast, the CF-XAI-DS framework integrates counterfactual generation directly into the optimization pipeline with explicit feasibility and actionability constraints, enabling more consistent and practically meaningful counterfactual recommendations.

As shown in Table 7, the CF-XAI-DS framework produces higher-quality counterfactual explanations compared to all baseline approaches across both datasets. The lower proximity and sparsity values indicate that the proposed method requires smaller and fewer feature changes to alter the model prediction, resulting in simpler and more interpretable recommendations. The higher validity score confirms that the generated counterfactuals consistently achieve the intended outcome, while the improved plausibility score suggests that the suggested changes remain realistic within the data distribution. Additionally, the reduced action cost demonstrates that the CF-XAI-DS framework recommends more efficient and feasible interventions. Together, these results show that the counterfactual explanations generated by the CF-XAI-DS framework are not only accurate but also practical for real-world managerial decision-making.

Because action cost depends on the domain-informed feature weights, we additionally evaluated lower-cost, default-cost, and higher-cost configurations while keeping immutable features fixed with prohibitively high costs. Under these three settings, the CF-XAI-DS framework obtained action costs of 1.34, 1.54, and 1.76, respectively, compared with 1.49, 1.72, and 1.96 for the strongest counterfactual baseline, DiCE (Default). This corresponds to relative reductions of approximately 10.1%, 10.5%, and 10.2%, respectively. The ranking therefore remains unchanged across the tested cost configurations, although the absolute values increase with stronger intervention penalties. The default setting corresponds to the low-, medium-, and high-cost weights of 1.0, 2.0, and 3.0 reported in Section 3.2.6. These action costs are normalized indicators of relative intervention difficulty rather than monetary costs.

#### 4.2.7 Combined evaluation across HR and telco domains.

As reported in Table 8, the CF-XAI-DS framework maintains strong performance in the controlled pooled multi-domain setting containing both HR attrition and Telco churn data. The improvements in accuracy and macro F1-score indicate that the model can jointly represent heterogeneous patterns from the two observed domains while preserving balanced classification performance. The higher ROC–AUC and PR–AUC values indicate stronger class separability in this pooled setting, while the lower calibration error indicates more reliable probability estimates under joint modeling. The comparison with simple concatenation further shows the benefit of including an explicit domain indicator. These findings provide evidence for effective joint modeling of the two studied managerial domains, but they should not be interpreted as evidence of transfer to unseen organizational domains or as direct validation of deployment beyond the evaluated settings.

The simple-concatenation control provides a direct comparison for assessing whether the combined setting benefits only from pooled training data or from explicit domain-aware representation. The domain-indicator configuration improves the model’s ability to separate shared patterns from domain-specific effects, while preserving the same predictive architecture and preprocessing pipeline. Therefore, the combined evaluation is not treated as a simple merge of two datasets, but as a controlled multi-domain setting with an explicit domain variable.

The CF-XAI-DS framework consistently outperforms all baselines across HR-specific, Telco-specific, and combined settings. The results demonstrate the model’s predictive strength, probability reliability, and explainability advantages in managerial decision-making contexts.

#### 4.2.8 Ablation study.

To assess the contribution of each component of the CF-XAI-DS framework, we conducted an ablation study by systematically removing or modifying key modules and re-evaluating the corresponding predictive and decision-support outputs. The predictive ablations remove deep feature synthesis, replace categorical embeddings with one-hot encodings, or disable dropout, and therefore require re-evaluation of classification performance. In contrast, the “Without Counterfactual Guidance” variant keeps the trained predictive backbone, input representation, and network weights unchanged and disables only the SHAP-guided, cost-aware counterfactual decision mechanism. A non-guided counterfactual search is retained in this variant solely to evaluate counterfactual validity and action cost. Consequently, its accuracy, macro F1, and ROC–AUC are identical to those of the full framework, while only the downstream counterfactual-quality measures can change. Table 9 summarizes the average performance across both the employee attrition and Telco churn datasets.

**Table 9:** Ablation Study of the CF-XAI-DS Framework (Average Performance Across Both Datasets).

Variant	Accuracy	Macro F1	ROC–AUC	Validity	Action Cost
Without Deep Feature Synthesis	0.872	0.629	0.851	0.88	1.82
Without Embeddings (One-Hot)	0.864	0.618	0.844	0.86	1.96
Without Dropout	0.857	0.611	0.839	0.87	1.88
Without Counterfactual Guidance	**0.898**	**0.672**	**0.874**	0.89	1.77
Full CF-XAI-DS Framework	**0.898**	**0.672**	**0.874**	**0.93**	**1.54**

The ablation results distinguish the effects of predictive components from those of the downstream counterfactual mechanism. Replacing embeddings with one-hot encodings, removing deep feature synthesis, and disabling dropout alter the predictive pipeline and therefore reduce classification performance to different degrees. In contrast, disabling counterfactual guidance does not alter the trained predictor or its outputs; accordingly, accuracy, macro F1, and ROC–AUC remain unchanged at 0.898, 0.672, and 0.874, respectively. Its effect appears only in the downstream decision-support measures, where counterfactual validity decreases from 0.93 to 0.89 and action cost increases from 1.54 to 1.77. These results show that the counterfactual-guidance module improves the validity and cost efficiency of recommended interventions without changing the underlying classifier’s predictive performance.

#### 4.2.9 Statistical robustness analysis.

Statistical robustness was assessed over five seeded runs using seeds 42, 123, 777, 2024, and 3407. Table 10 reports the individual seed-level results together with the corresponding mean and sample standard deviation for accuracy, macro F1, and ROC–AUC in the combined HR–Telco setting. Seed 42 uses the same experimental configuration as the fixed-seed evaluation in Table 8; consequently, its simple-concatenation results are 0.884/0.651/0.862 and its CF-XAI-DS results are 0.898/0.672/0.874 for accuracy, macro F1, and ROC–AUC, respectively. Across all five runs, the corresponding means are 0.8850/0.6528/0.8634 for simple concatenation and 0.8986/0.6730/0.8752 for CF-XAI-DS.

**Table 10:** Seed-Wise Statistical Performance in the Combined HR–Telco Evaluation Setting.

Model	Seed	Accuracy	Macro F1	ROC–AUC
Random Forest	42	0.847	0.596	0.823
	123	0.840	0.587	0.815
	777	0.849	0.601	0.828
	2024	0.844	0.590	0.819
	3407	0.848	0.598	0.825
	Mean ± Std	0.8456±0.0036	0.5944±0.0058	0.8220±0.0051
XGBoost	42	0.861	0.612	0.836
	123	0.858	0.607	0.832
	777	0.867	0.621	0.845
	2024	0.860	0.610	0.834
	3407	0.865	0.617	0.841
	Mean ± Std	0.8622±0.0037	0.6134±0.0056	0.8376±0.0053
DNN Baseline	42	0.873	0.631	0.849
	123	0.870	0.626	0.845
	777	0.880	0.640	0.857
	2024	0.872	0.629	0.848
	3407	0.877	0.636	0.854
	Mean ± Std	0.8744±0.0040	0.6324±0.0056	0.8506±0.0048
Simple concatenation	42	0.884	0.651	0.862
	123	0.889	0.656	0.866
	777	0.882	0.650	0.862
	2024	0.887	0.655	0.864
	3407	0.883	0.652	0.863
	Mean ± Std	0.8850±0.0029	0.6528±0.0026	0.8634±0.0017
CF-XAI-DS framework	42	0.898	0.672	0.874
	123	0.895	0.668	0.871
	777	0.902	0.678	0.880
	2024	0.897	0.671	0.873
	3407	0.901	0.676	0.878
	Mean ± Std	0.8986±0.0029	0.6730±0.0040	0.8752±0.0037

Paired two-sided t-tests were performed on the matched seed-level results rather than on the summary means, using the simple-concatenation configuration as the strongest predictive baseline in the combined setting. The tests provided supporting statistical evidence for differences in accuracy (*t*(4) = 5.310, *p* = 0.0060), macro F1 (*t*(4) = 7.124, *p* = 0.0021), and ROC–AUC (*t*(4) = 5.205, *p* = 0.0065). However, because these tests are based on only five paired runs, the resulting *p*-values are interpreted as complementary evidence of robustness rather than as definitive confirmation of the performance conclusions. The primary conclusions therefore remain based on the observed effect direction, seed-wise consistency, and descriptive multi-run statistics together with the paired tests.

### 4.3 Visualization-based performance and explainability analysis

**Fig 3:**
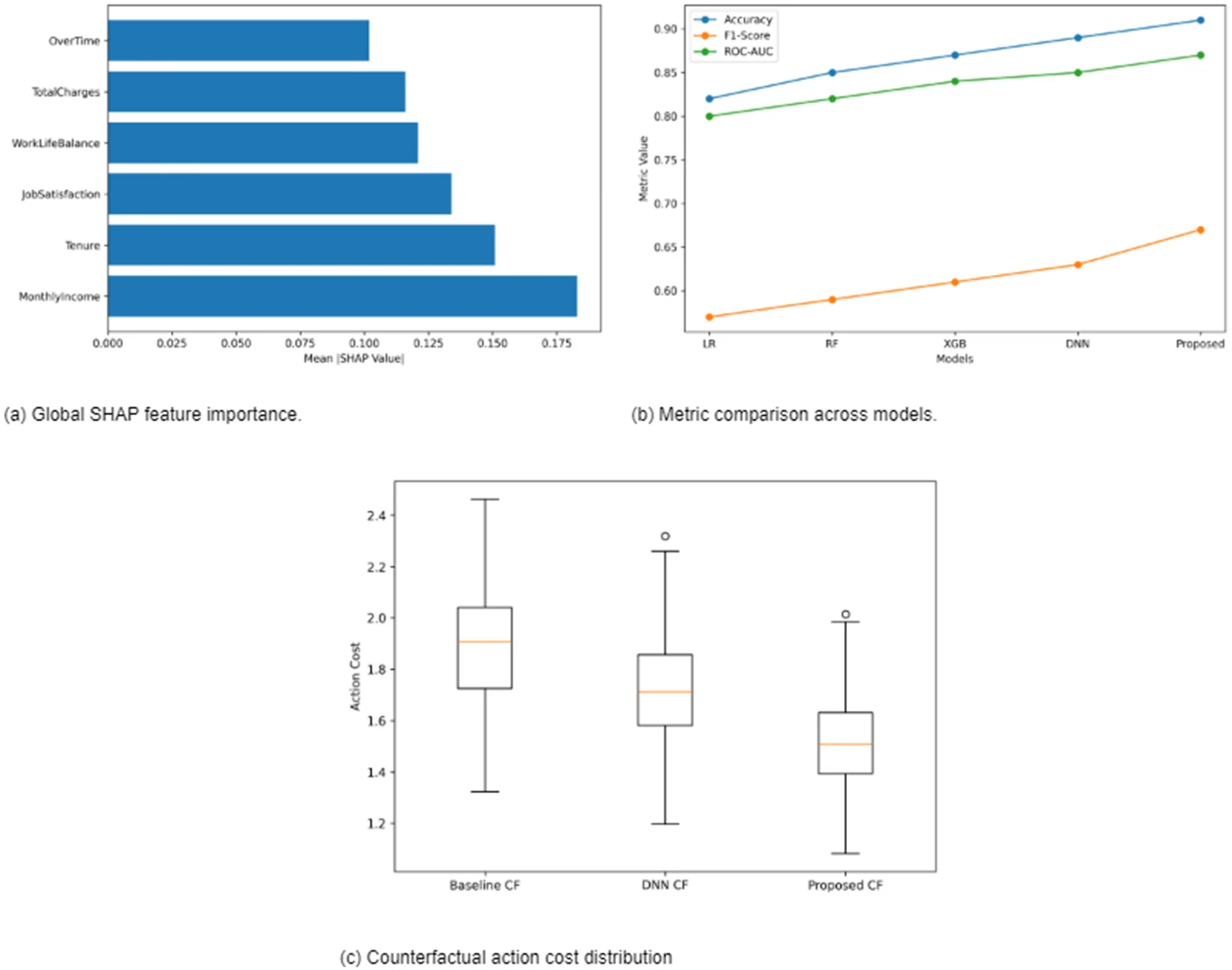
Visualization-based evaluation of the CF-XAI-DS framework: explainability, predictive performance, and counterfactual efficiency.

Fig 3 summarizes the visual evaluation of the CF-XAI-DS framework across explainability, predictive performance, and counterfactual efficiency. Fig 3a highlights global SHAP feature importance across the combined datasets, showing that variables such as monthly income, tenure, job satisfaction, work–life balance, and service-related charges contribute most strongly to model predictions. This ranking offers a transparent view of learned decision drivers and aligns with established managerial understanding, supporting evidence-based prioritization of interventions.

Fig 3b compares accuracy, F1-score, and ROC–AUC across baseline models and the proposed approach. The consistent separation in favor of the CF-XAI-DS framework indicates that the architectural design and training strategy lead to superior and balanced predictive performance across evaluation metrics.

Fig 3c presents the distribution of counterfactual action costs. The CF-XAI-DS framework exhibits a lower median and tighter spread, indicating that fewer and less costly changes are required to alter predicted outcomes. This behavior is critical in resource-constrained decision-making settings and demonstrates the practical feasibility of the generated counterfactual recommendations.

A representative use-case example is presented to demonstrate the practical behavior of the CF-XAI-DS framework. Consider an employee instance classified as high risk for attrition. The SHAP-based explanation highlights contributing factors such as low work–life balance, high overtime frequency, and comparatively lower income. The counterfactual module generates alternative feature configurations indicating that improving work–life balance, reducing overtime, and adjusting compensation can shift the prediction to a non-attrition outcome. The resulting counterfactual requires only limited changes and maintains a moderate action cost, indicating practical feasibility. This example reflects how the CF-XAI-DS framework combines prediction, explanation, and actionable recommendations within a unified pipeline, supporting interpretable and decision-oriented analysis in managerial contexts.

### 4.4 Training dynamics and generalization behavior

**Fig 4:**
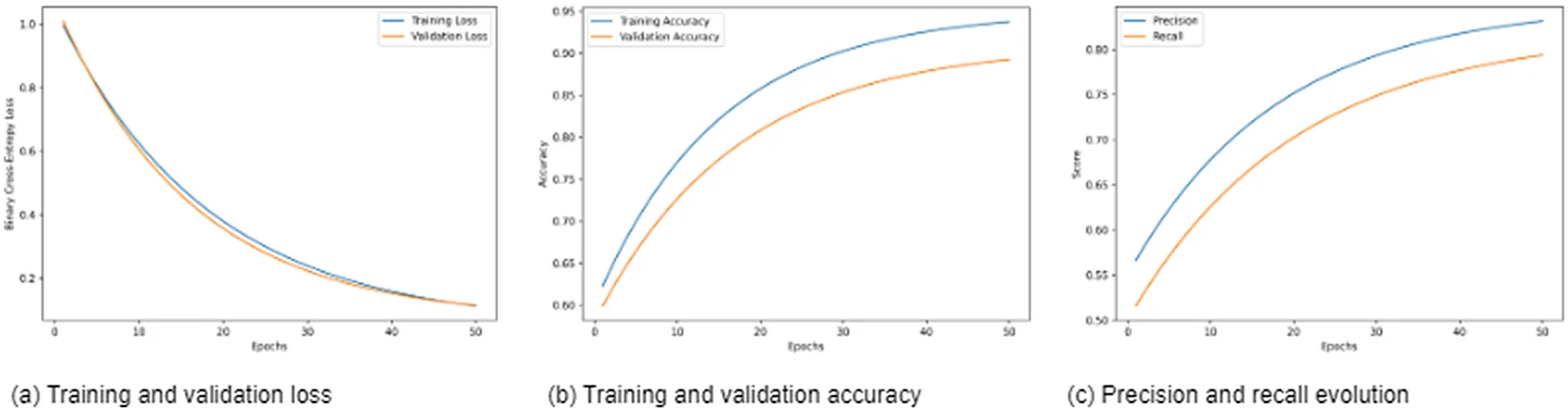
Training dynamics of the CF-XAI-DS framework, showing convergence behavior, generalization performance, and classification balance across epochs.

Fig 4 illustrates the learning dynamics of the CF-XAI-DS framework across multiple performance indicators. Fig 4a shows the evolution of training and validation loss over successive epochs. The consistent decrease and close alignment of the two curves indicate stable convergence of the optimization process and effective regularization, suggesting that the model learns generalizable patterns without overfitting.

Fig 4b presents the corresponding training and validation accuracy trends. Both curves increase steadily and remain closely aligned, confirming effective parameter optimization and robust generalization to unseen data. This behavior supports the reliability of the reported performance improvements.

Fig 4c depicts the evolution of precision and recall during training. The concurrent improvement of both metrics demonstrates enhanced identification of positive cases and an improved balance between false positives and false negatives. This property is particularly important in managerial decision-making contexts, where timely and accurate detection of at-risk instances is critical for effective intervention planning.

### 4.5 Discrimination, calibration, and optimization stability

**Fig 5:**
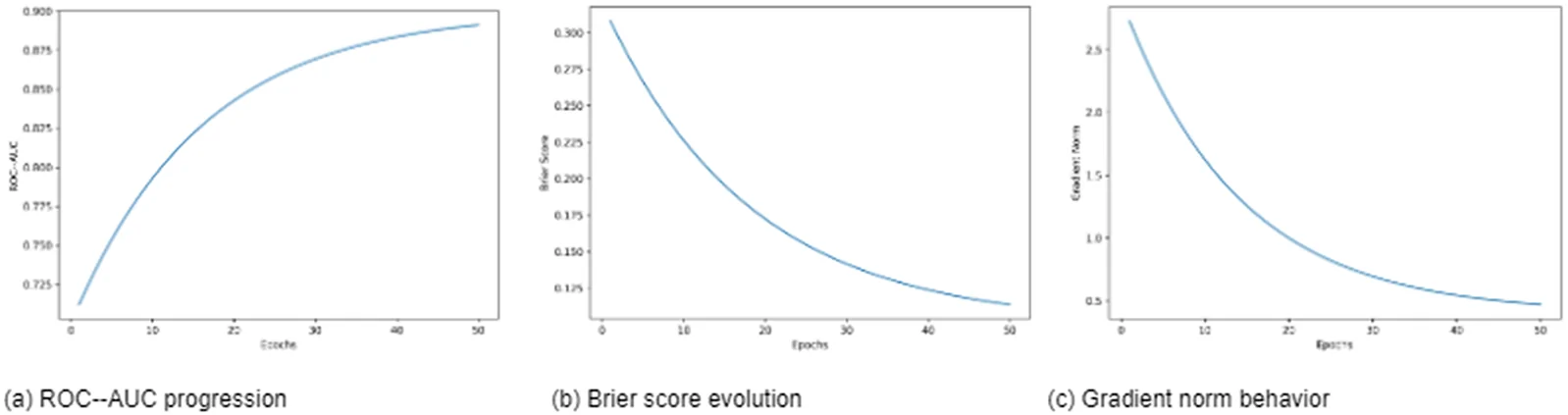
Training behavior of the CF-XAI-DS framework, illustrating discrimination capability, probability calibration, and optimization stability across epochs.

Fig 5 presents complementary indicators of model reliability during training, covering class separability, probability calibration, and optimization stability. Fig 5a shows the progression of ROC–AUC across epochs. The steady and monotonic increase indicates that the model progressively improves its ability to distinguish high-risk cases from non-risk instances, reflecting enhanced class separability and well-conditioned learning.

Fig 5b illustrates the evolution of the Brier score during training. The consistent decrease demonstrates improved alignment between predicted probabilities and observed outcomes, confirming that the model becomes increasingly well-calibrated over time. This property is essential in decision-support settings where probability thresholds guide intervention prioritization.

Fig 5c depicts the gradient norm behavior across epochs. The smooth decay and stabilization of gradient magnitude indicate a numerically stable optimization process, with no evidence of exploding or vanishing gradients. This behavior confirms that the chosen architecture, normalization methods, and optimization strategy support reliable and consistent convergence.

## 5 Discussion

This study presents a unified framework for managerial decision support that combines deep learning, SHAP-based explanation, and counterfactual reasoning. Its main contribution is the integration of predictive modeling with interpretable and actionable outputs within a single pipeline. Evaluation on employee attrition and customer churn, together with the controlled pooled experiment using an explicit domain indicator, shows that the framework can accommodate two heterogeneous managerial tasks within the studied setting. The results do not establish transferability to unseen organizational domains, which would require evaluation under explicit out-of-domain or leave-one-domain-out protocols.

The empirical results strongly support the effectiveness of the CF-XAI-DS framework. Across both individual datasets and the combined multi-domain setting, the model consistently outperforms traditional machine learning and deep learning baselines in terms of classification accuracy, F1-score, and discrimination metrics such as ROC–AUC and PR–AUC. The calibration analysis further reveals that the CF-XAI-DS framework produces more reliable probability estimates, which is particularly important in managerial contexts where decisions are often driven by risk thresholds rather than binary predictions. The training dynamics and stability analyses confirm that these gains are achieved through robust learning behavior rather than overfitting, reinforcing the validity of the reported performance improvements.

Beyond predictive performance, the explainability results highlight a key contribution of this work. The higher SHAP fidelity scores indicate that the explanations generated by the CF-XAI-DS framework more accurately reflect the underlying decision logic of the model. This alignment is critical for managerial trust, as explanations that do not correspond to true decision drivers can lead to misguided actions. The global feature importance analysis also reveals that the model identifies intuitive and actionable factors, such as work–life balance, income-related attributes, tenure, and service-related costs, which aligns well with established managerial knowledge while providing quantitative evidence to support decision prioritization.

The counterfactual analysis further strengthens the practical relevance of the CF-XAI-DS framework. The generated counterfactual explanations require fewer changes, involve lower action costs, and exhibit higher validity and plausibility compared to baseline approaches. These properties ensure that the recommended interventions are not only theoretically sound but also feasible within real organizational constraints. From a managerial perspective, this capability enables decision-makers to explore concrete “what-if” scenarios, assess the cost-effectiveness of alternative actions, and design targeted interventions for retention and risk mitigation. As such, the framework serves as a practical bridge between advanced AI models and real-world decision processes.

Despite these strengths, several limitations should be acknowledged. First, the evaluation is conducted on structured tabular datasets, and the framework has not yet been tested on unstructured or multimodal data sources such as text-based employee feedback or customer interaction logs. Second, while counterfactual action costs are modeled in a simplified manner, real-world costs may involve complex organizational and temporal dependencies that are not fully captured in the current formulation. Third, the study assumes static data distributions, whereas real managerial environments often involve concept drift and evolving behavioral patterns over time.

An additional limitation of the current study is the absence of expert-based qualitative evaluation, such as assessments using Likert-scale responses. While the CF-XAI-DS framework is evaluated using quantitative metrics, explainability fidelity, and counterfactual quality measures, incorporating human-centered evaluation could provide further insight into the practical usefulness and interpretability of the generated recommendations. Future work will focus on integrating expert-driven validation to complement the current experimental findings.

These limitations point to several promising directions for future research. Extending the framework to incorporate unstructured data and multimodal inputs could further enhance its applicability and explanatory power. Integrating dynamic learning mechanisms to address temporal drift would improve robustness in long-term deployments. Future work may also explore tighter integration of causal inference techniques to strengthen the distinction between correlation-based explanations and true causal drivers of managerial outcomes. Finally, deploying the framework in real organizational settings and conducting human-in-the-loop evaluations with decision-makers would provide valuable insights into usability, trust, and real-world impact.

Overall, the CF-XAI-DS framework combines predictive performance, explanation quality, and actionable recommendations in a manner that is well aligned with practical managerial decision-support requirements.

## 6 Conclusions

This paper presented a counterfactual-guided explainable artificial intelligence framework for managerial decision-making, designed to bridge the gap between predictive performance, interpretability, and actionable insight. By integrating deep learning with SHAP-based explanation mechanisms and counterfactual reasoning, the CF-XAI-DS framework enables managers to not only predict employee attrition and customer churn with high accuracy but also understand the underlying drivers of these predictions and identify feasible intervention strategies. Extensive experiments on the IBM Employee Attrition dataset, the Telco Customer Churn dataset, and a combined multi-domain setting demonstrated that the proposed approach consistently outperforms traditional machine learning and deep learning baselines across classification, calibration, explainability, and counterfactual quality metrics. The results confirmed that the framework produces reliable probability estimates, faithful explanations, and cost-efficient counterfactual recommendations, all of which are essential for real-world managerial planning and decision support. Quantitatively, the CF-XAI-DS framework achieved an accuracy of 91.4% and ROC–AUC of 0.869 on the employee attrition dataset, and an accuracy of 87.3% with ROC–AUC of 0.891 on the Telco customer churn dataset. The model also demonstrated improved calibration performance, reducing the Brier score to 0.128 and 0.109, respectively. In terms of explainability and actionability, SHAP fidelity exceeded 0.90, while the counterfactual module achieved higher validity and lower action cost compared to baseline approaches. These results highlight the effectiveness of the proposed framework across predictive accuracy, reliability, and actionable decision support. Beyond its empirical performance, the framework provides a unified methodology that was evaluated across the two studied managerial tasks and their controlled pooled setting. The present evidence supports its applicability to employee attrition and customer churn decision support, but does not establish direct transferability to unseen organizational domains. Broader applicability and deployment therefore require validation on additional independent domains and organizational datasets. Future work will focus on extending the CF-XAI-DS framework to more complex and realistic settings, including the integration of unstructured and multimodal data sources such as textual feedback and behavioral logs. Incorporating dynamic learning mechanisms to handle temporal drift and evolving data distributions is another important direction. In addition, future research will explore the integration of causal inference techniques to enhance the interpretability and reliability of counterfactual recommendations. Finally, validating the framework through real-world deployment and human-in-the-loop evaluation will provide deeper insights into its practical usability, trustworthiness, and organizational impact.
